# Ovarian tissue cryopreservation and transplantation: scientific implications

**DOI:** 10.1007/s10815-016-0814-1

**Published:** 2016-10-08

**Authors:** Sherman Silber

**Affiliations:** 1 Infertility Center of St. Louis, 224 South Woods Mill Road, Suite 730, Saint Louis, MO 63017 USA; 2 Sun Yat-Sen Medical School, Guangzhou, China

**Keywords:** Primordial follicle, Oocyte recruitment, Oocyte arrest, Ovary transplant, Cryopreservation, Fertility preservation, In vitro oogenesis

## Abstract

After fresh or frozen ovary transplantation, FSH levels return to normal, and menstrual cycles resume by 150 days, coincident with anti-Müllerian hormone rising to higher than normal levels. AMH then returns to well below normal levels by 240 days, remaining as such for many years with nonetheless normal ovulation and fertility. To date, 20 babies have been born in our program from 11 fresh and 13 cryopreserved ovary transplant recipients with a live baby rate of over 70 % (11 babies from fresh and 9 from frozen). Globally, over 70 live births have been reported for both fresh and frozen ovary transplants with an approximate 30 % live birth rate. Given the rapid rise of AMH after the fall of FSH, with a subsequent AMH decrease with retention of ovarian function, it is tempting to speculate the existence of a shared mechanism controlling primordial follicle recruitment, fetal oocyte meiotic arrest, and recruitment in the adult ovary. With the massive recruitment of primordial follicles observed after human ovarian cortical tissue transplantation, which subsides to an extremely low recruitment rate, we will discuss how this phenomenon suggests a unifying theory implicating ovarian cortical tissue rigidity in the regulation of both fetal oocyte arrest and recruitment of follicles in the adult ovary. As the paper by Winkler-Crepaz et al. in this issue demonstrates, our in vivo results are consistent with the in vitro demonstration that primordial follicles in the fetal cortex are “locked” in development, resulting in meiotic arrest, which spares the oocytes from being rapidly lost all at once (Winkler-Crepaz et al., *J Assist Reprod Genet*, 1). Winkler-Crepaz et al. demonstrate that follicle loss after ovarian cortex transplantation is unlikely due to ischemic apoptosis, but rather from a “burst” of primordial follicle recruitment. In vivo, primordial follicles are normally resistant to further development or activation to prevent oocyte depletion. The dense fibrous ovarian cortex, through as yet unresolved mechanisms, arrests the further continuation of meiosis and also prevents a sudden depletion of all resting follicles in the adult ovary. Intrinsic tissue pressure is released after cortical tissue transplantation, temporarily resulting in a rapid follicle depletion. These results are consistent with the observation that once the ovarian reserve is reduced in the graft, the rate of recruitment diminishes and the ovarian tissue exhibits a relatively long duration of function.

## Introduction

The developed world is in the midst of a widespread infertility epidemic. Economies in Japan, the USA, southern Europe, and even China are threatened by a decreasing population of young people having to support an increasing population of elderly and retirees [1]. Infertility clinics are popping up throughout the world in huge numbers because of a worldwide decline in fertility as women age and become less fertile [2]. In her teen years, a woman has a 0.2 % chance of being infertile, and by her early twenties, it is up to 2 %. By her early thirties, it is up to 20 % [2, 3]. Many modern women today do not consider having children until their mid-thirties, by which time nearly 20 % are infertile, simply due to the age-related decline in the number and quality of their oocytes. This is clearly demonstrated by the high pregnancy rate using donor oocytes from young women placed into the uterus of older women [2–12].

As important for reproductive medicine as is aging of the population and the subsequent worldwide epidemic of infertility, is the high incidence of cancer in girls and young women, curable in the majority of cases at the cost of rendering them sterile. Almost 6 % of women of reproductive age are cancer survivors. They will eventually have been sterilized by their chemotherapy or radiation [13–21].

Until recently, oocyte freezing had very poor to no success, and thus ovary tissue slow freezing was the only cryopreservation method we could rely upon [22–24]. More commonly now, vitrification is used instead of slow freeze for oocyte cryopreservation [25–34]. However, many programs do not have follow-up results with oocyte freezing especially in cancer patients undergoing sterilizing chemotherapy and radiation. Furthermore, it may require several cycles of ovarian stimulation to obtain enough oocytes to give women some level of comfort, because even with fresh oocytes, there is only a 5 % pregnancy rate per egg [35].

As an alternative strategy for cancer patients, ovarian tissue freezing has benefits over egg freezing. In some patients, freezing ovarian tissue obviates the need to delay treatment for a stimulation cycle. Furthermore, transplanting ovarian tissue not only restores fertility but also restores endocrine function.

As important as these clinical benefits afforded by ovary cryopreservation and transplantation are, there may be even more important basic science implications to be learned about the ovary, as is evident in the current paper by Winkler et al., as well as recent papers by Silber et al. and by Hayashi et al. [36–42]. The purposes of this review are to explain the clinical employment of ovary freezing and transplantation and to delineate what we have learned about primordial follicle arrest and recruitment.

## Ovarian cortex cryopreservation and transplantation

**Fig. 1:**
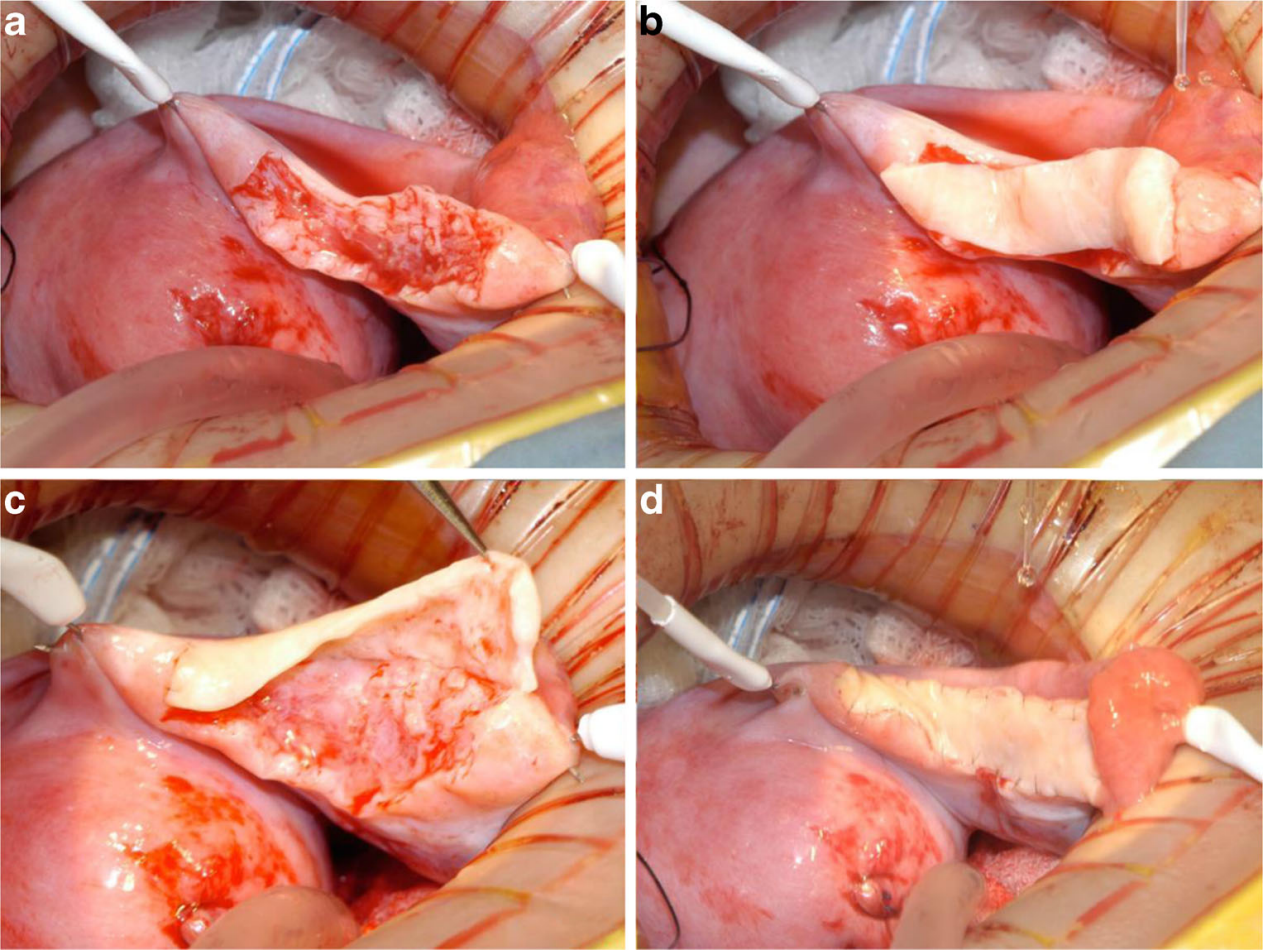
Steps in the procedure of ovarian transplantation between MZ twin sisters: **a** preparation of donor ovarian cortex by dissection in a Petri dish on ice; **b** preparation of recipient ovarian medulla; **c** attaching donor cortical tissue to recipient ovarian medulla; **d** attaching thawed donor cortical tissue for retransplant to the recipient medulla [37]

Cryopreservation and transplantation of ovarian tissue has a long history in animal studies and in early human studies. In 1960, Parrott and colleagues showed that ovarian tissue could be successfully frozen and autografted in mice, and similar studies by Gunasena and colleagues 37 years later verified live births of mice after autologous transplantation of cryopreserved mouse ovaries, originally shown in rats in 1954 [43–45]. Others have shown that mice have a normal reproductive lifespan after autografts of fresh tissue [46]. Researchers in the 1990s showed that in both mice and sheep, frozen ovarian tissue could be successfully thawed and autotransplanted leading to normal ovarian function and live births [22, 47]. We reported in 2004 the first live birth from fresh human ovarian tissue transplanted between identical twins discordant for premature ovarian failure [37] (Fig. 1a–d). Donnez and colleagues reported what is deemed to be the first human live birth from orthotropic transplantation of frozen human ovarian tissue in 2004, with another successful live birth achieved by Meirow in 2005 [48, 49].

**Fig. 2:**
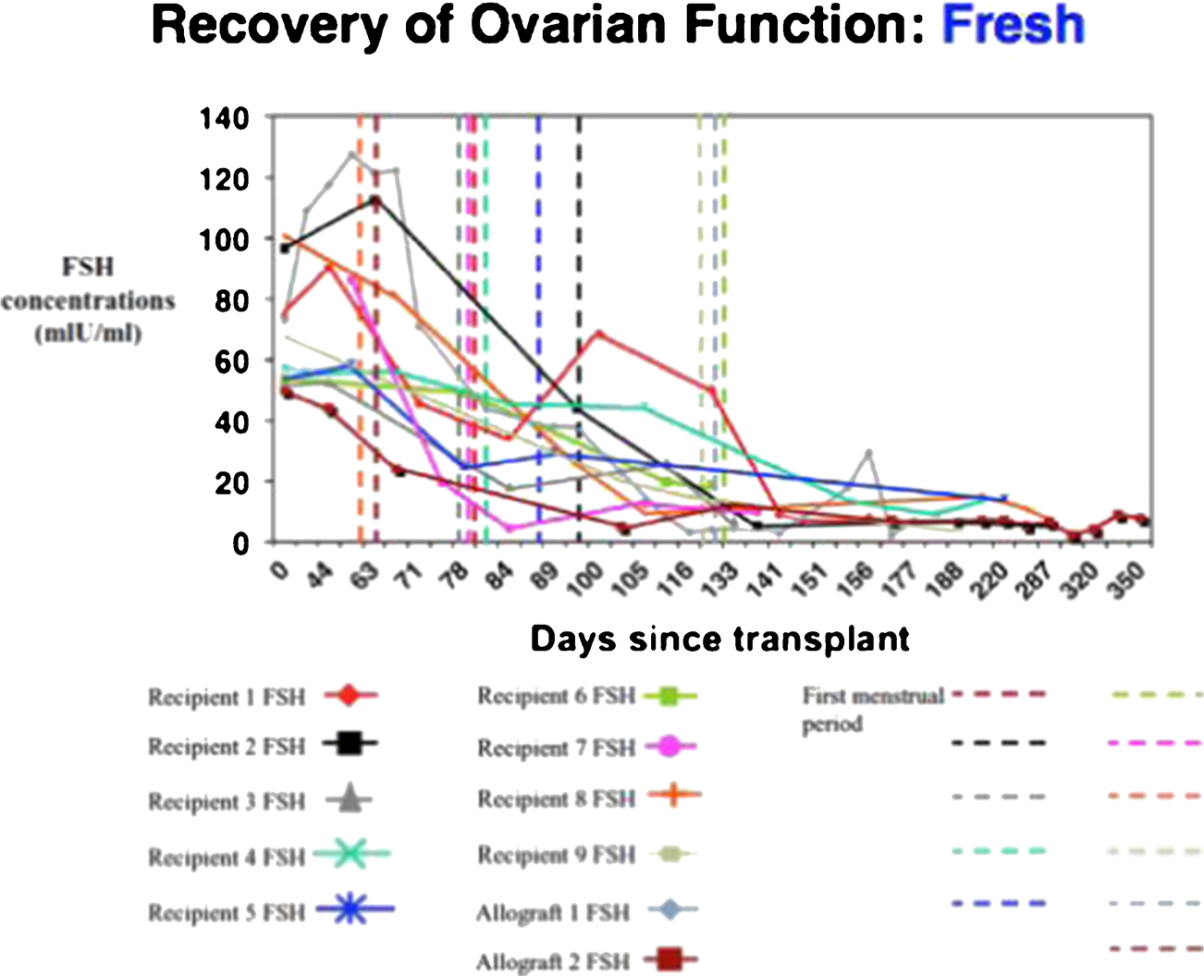
Serum FSH returns to normal consistently by 4.5 months after fresh transplant [39]

**Fig. 3:**
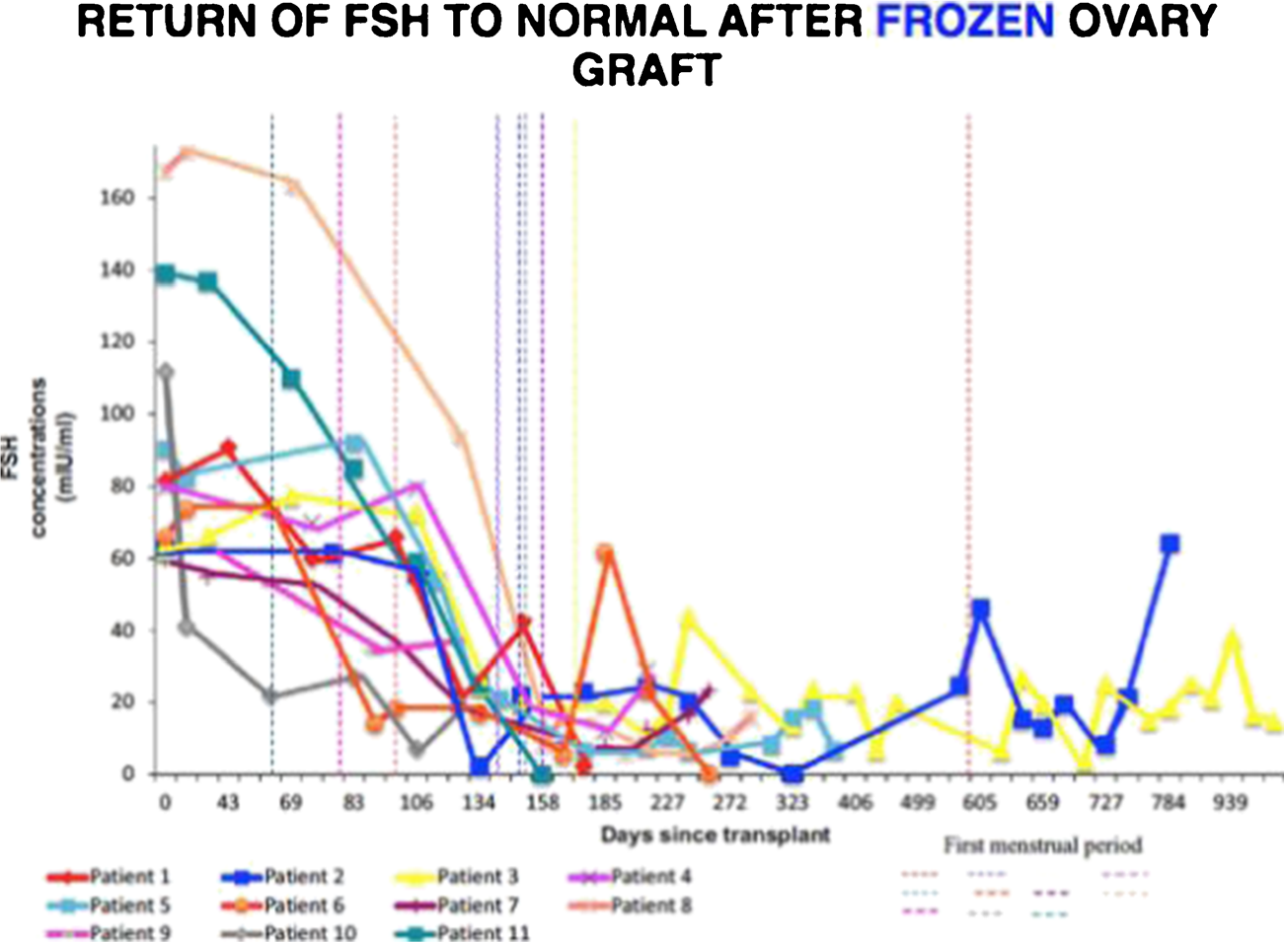
Similar to fresh transplant, the FSH returns to normal about 5 months after frozen transplant [39]

**Fig. 4:**
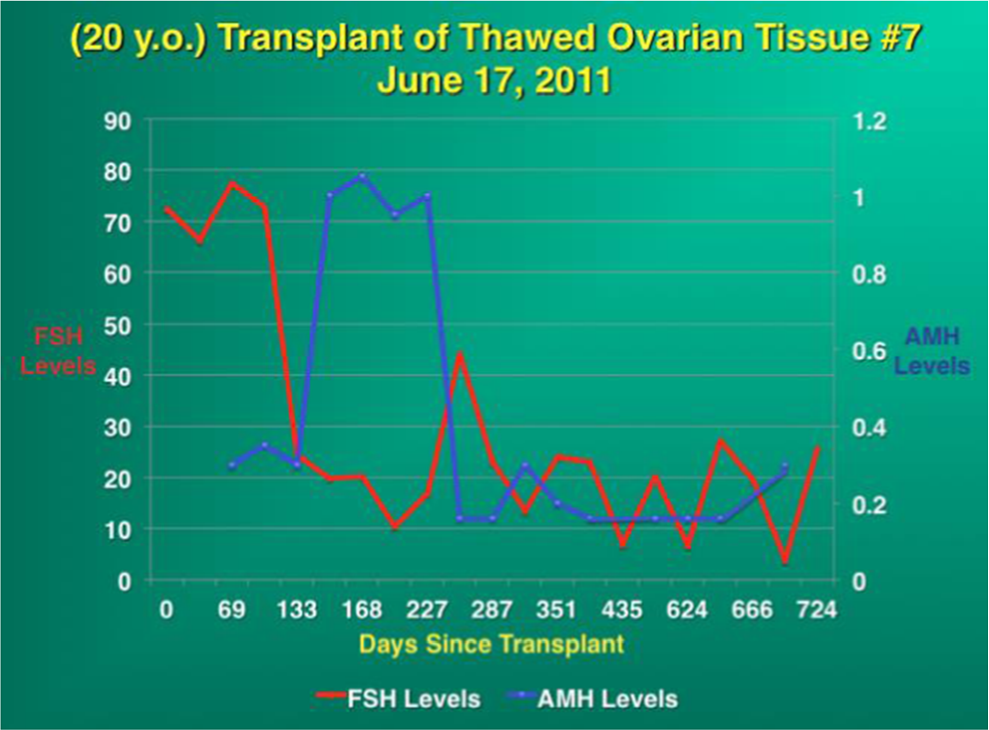
As the FSH returns to normal, the AMH rises very high and then goes down to very low levels

**Fig. 5:**
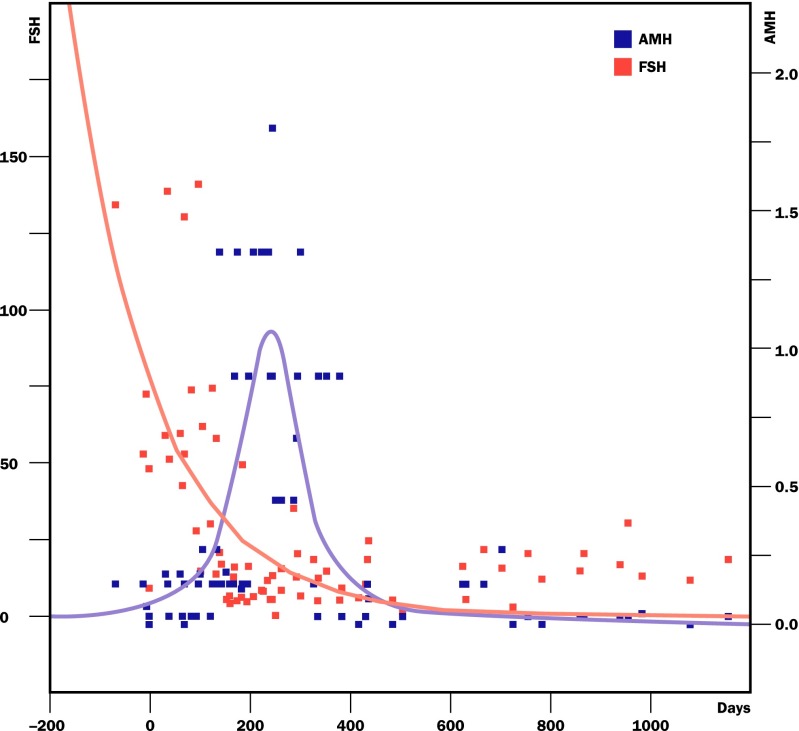
Composite dot graph summarizing the return of FSH to normal and the rise of AMH and subsequent decline after frozen transplant [38]

Our large series of 11 fresh ovary transplants resulted in 14 pregnancies and 11 healthy babies, and a remarkably consistent return of menstrual cycling and normal day 3 follicle-stimulating hormone (FSH) concentrations by 4 to 5 months in all patients, which gave hope that a large series of cryopreserved transplants might also provide robust results [39, 50–52]. In fact, the use of similar surgical techniques with cryopreserved ovarian tissue for patients with cancer led to 10 pregnancies, and 9 healthy babies from 13 cryopreserved transplants, in addition to the 14 pregnancies after fresh ovarian cortex transplants between twins (11 live babies) for a total of 20 healthy babies. Our unusual series of fresh and frozen ovary transplants allowed us to evaluate the effect, if any of cryopreservation versus the transplant itself. In fact, cryopreservation had no significant impact on ovarian reserve, but over-recruitment of primordial follicles did (Figs. 2 and 3). The finding that as FSH decreases and ovulation resumes, with AMH initially rising to high levels followed by a return to very low levels, indicated that a massive over-recruitment of primordial follicles led to a subsequent depletion in the ovarian reserve. Interestingly, despite low AMH levels, the grafts nonetheless sustained ovarian function for long periods of time (Figs. 4 and 5).

The question was raised of what effect ovary donation might have on the donors’ reproductive lifespan. This question is not just an ethical one. It impacts the whole scientific issue of primordial follicle recruitment rate related to a decrease in ovarian reserve. The potential effect of unilateral oophorectomy on both fertility and age of onset of menopause is crucial for understanding how these ovarian grafts can last so long despite a very low AMH. Gosden and colleagues in 1989 described a compensatory mechanism of follicle rescue in mice that prevented any major effect on fertility [53]. A later study in 1992 noted that long-term ovarian function is not substantially affected by reduction of ovarian mass [54]. Other more clinical papers also support a lack of major effect on fertility of unilateral oophorectomy in human beings, with menopause occurring only 1 or 2 years earlier than in controls. Only one study has disputed this view in rats and suggested an earlier onset of menopause after unilateral oophorectomy than in controls [55–63]. However, if the traditional view is correct, in which unilateral oophorectomy does not negatively affect fertility and does not hasten the onset of menopause, this would support partial or complete oophorectomy and ovarian tissue cryopreservation to expand the reproductive lifespan of normal women who wish to delay childbearing but do not want to lose their current reproductive potential. Thus, we felt comfortable in undertaking a series of fresh ovary transplants, which led the way toward improving our ovarian freezing transplantation methods [64–66]. Also, we feel confident that removing an ovary will not harm long-term fertility. Contrarily, it follows that transplanting the removed ovary could extend a woman’s reproductive lifespan.

Several techniques have been described for transplantation of the ovarian cortex [37, 43, 48–50]. In mice, Parrot used sliced little pieces of ovarian cortex. Others prepared peritoneum near the ovary but then switched to a technique similar to that described for fresh ovarian tissue [37]. Ovarian cortical slices can also be transplanted under the surface of the cortex in the nonfunctional ovary [49]. All these techniques have resulted in babies and there is no consensus on which is best.

**Fig. 6:**
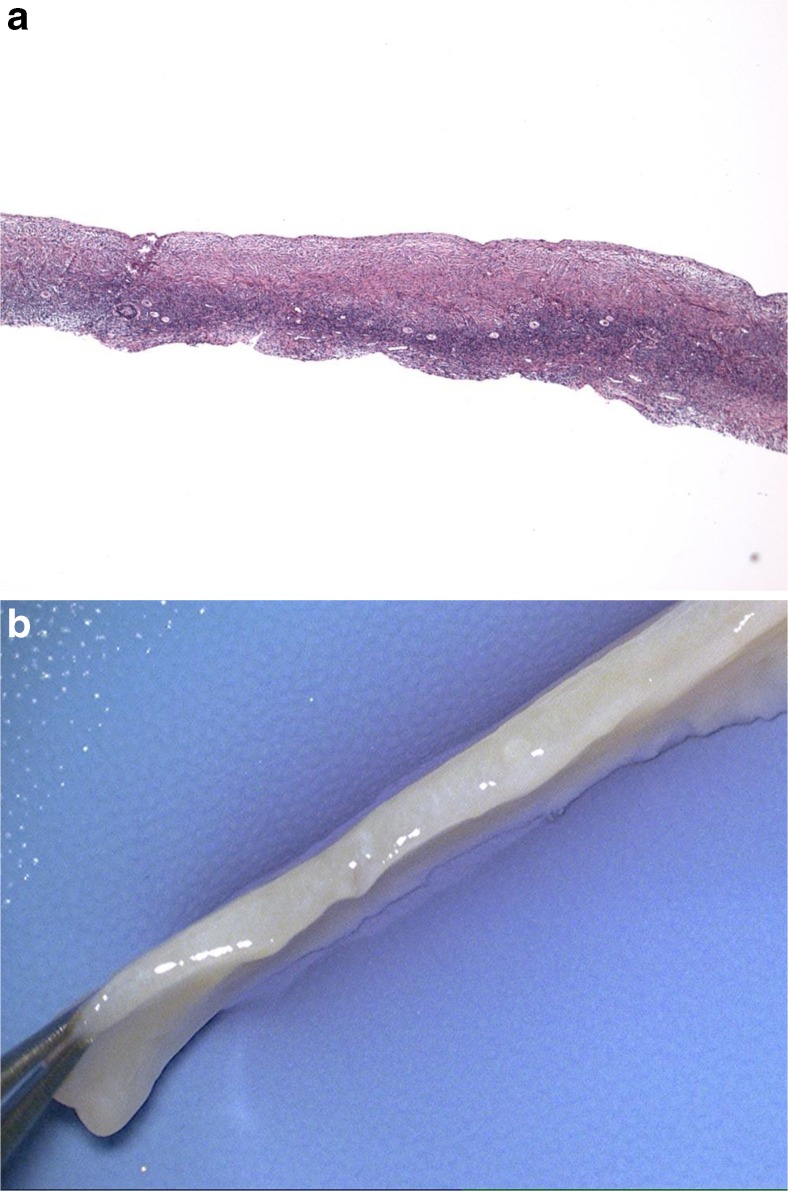
**a**, **b** All of the resting follicles are located in the outer 1 mm of the fibrous ovarian cortex

The key to successful transplantation of frozen ovarian tissue is to consider it as though it were a skin graft (Fig. 6a, b). Microhematoma formation under the graft is avoided by microbipolar cautery pulsatile irrigation and micropressure stitches of 9–0 nylon. Constant pulsatile irrigation with heparinized saline prevents adhesions, improving chances of spontaneous pregnancy with no need for IVF, difficult in these cases due to a reduced ovarian reserve yielding few oocytes after ovarian hyperstimulation. The transplant is best if orthotropic, and adhesions are minimized [67, 68] (Fig. 1a–d).

Initially, there were only a few case reports, some very recently, of successful cryopreserved ovary transplantation but no unified single series [68–76]. However, it appears now that there is a worldwide live birth rate of over 30 to 70 %, with more than 70 babies, and long-term function of the transplant has been observed despite very low AMH. Robust results are seen in series from St. Louis, Brussels, Paris, Spain, Denmark, Israel, Japan, Italy, Germany, Australia, and Russia [77, 78]. Cryopreserved ovarian tissue grafts with the slow-freezing method as well as with vitrification are functional for more than 5 years, and many spontaneous pregnancies have been reported with no need for in vitro fertilization or other ancillary treatment. Most pregnancies were achieved without the need for in vitro fertilization and resulted instead from regular intercourse with no other treatment.

With this in mind, the remarkable long-term hormonal and ovulatory function of these cortical grafts despite extremely low AMH after the initial primordial follicle over-recruitment emphasizes the compensatory relationship of a low remaining ovarian reserve to a slower recruitment rate of primordial follicles [73].

The most common benefit of ovarian transplantation was previously thought to be the preservation of fertility and future endocrine function in young women undergoing cancer treatment. However, in the absence of pelvic irradiation for cancer treatment, why not use ovarian tissue cryopreservation in otherwise healthy women who wish to preserve their fertility for nonmedical reasons? With vitrification methods, there is no difference in the viability or integrity of cryopreserved ovarian tissue compared with fresh ovarian tissue [66]. Furthermore, with cryopreserved ovarian tissue transplantation, hormonal function is restored in addition to fertility [73–77].

## Ovarian cryopreservation techniques

In the past, all of the frozen ovary cases transplanted back to the patient have utilized the slow freeze approach [22–24, 79]. However, we now use vitrification exclusively for cryopreservation in humans because of the results of in vitro viability analysis in humans, as well as in vivo transplant studies in the bovine and human [65, 66]. Three of our successful eight pregnancies were from vitrified ovarian tissue. Five were from ovarian tissue that was frozen long ago (as early as 1996) with slow freeze.

**Fig. 7:**
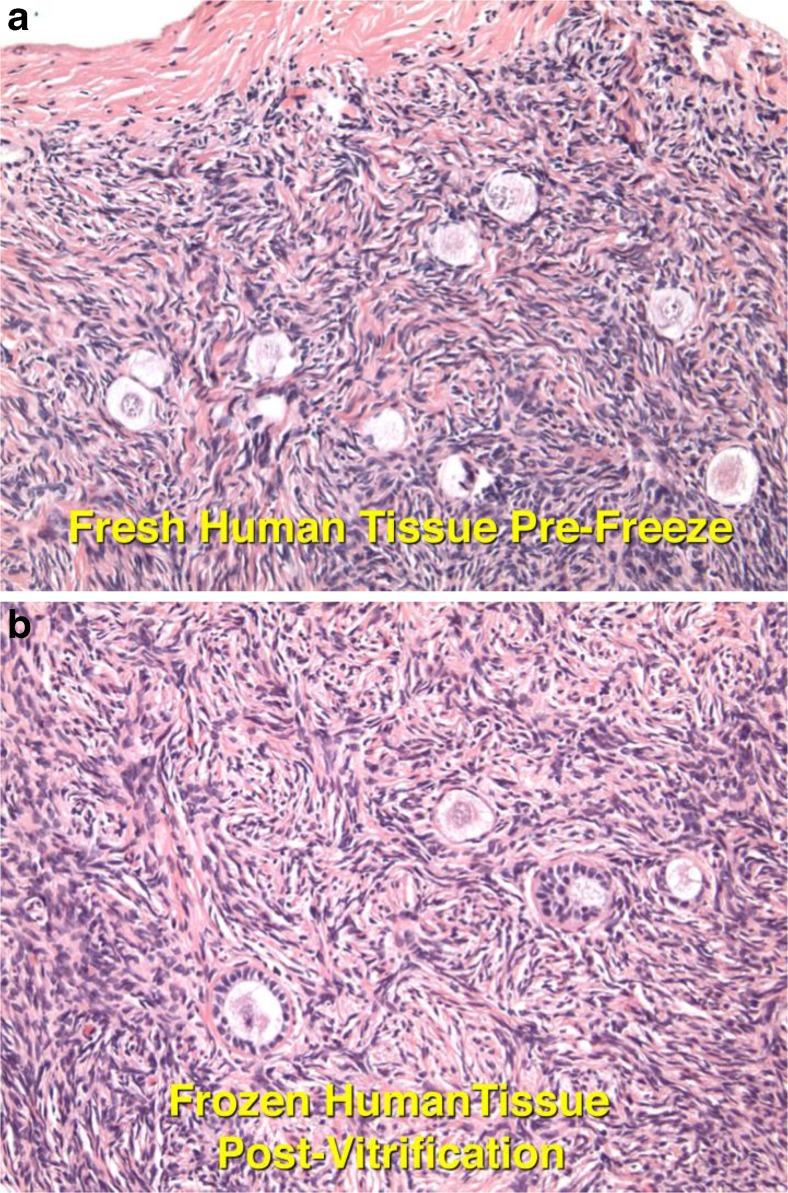
**a**, **b** There is no discernible difference between fresh ovarian tissue and vitrified [66]

The high viability (92 %) of oocytes in control (fresh) and vitrified specimens indicates virtually no damage to the eggs from ovarian tissue vitrification [66]. Overall, 2301 oocytes were examined from 16 specimens. There was no significant difference between fresh and vitrified tissue, but the viability of slow freeze-cryopreserved tissue was less than one half that of vitrified tissue or controls (42 %) (*P* < 0.01) (Fig. 7a, b). Transmission electron microscopy also has been used to analyze ovarian tissue that had been either cryopreserved by slow freezing or vitrified by ultra-rapid freezing, showing vitrification to be superior [64]. Standard H&E histology showed no difference between prefreeze ovarian tissue and postvitrification ovarian tissue. Finally, quantitative histologic study of primordial follicles in the bovine after vitrification and transplantation back to the cow 2 months later remarkably showed no follicle loss [65]. Nonetheless, for clinical use, slow freeze gives pregnancy results as good as vitrification. The only advantage of vitrification, and why we prefer it, is the ease of use.

**Fig. 8:**
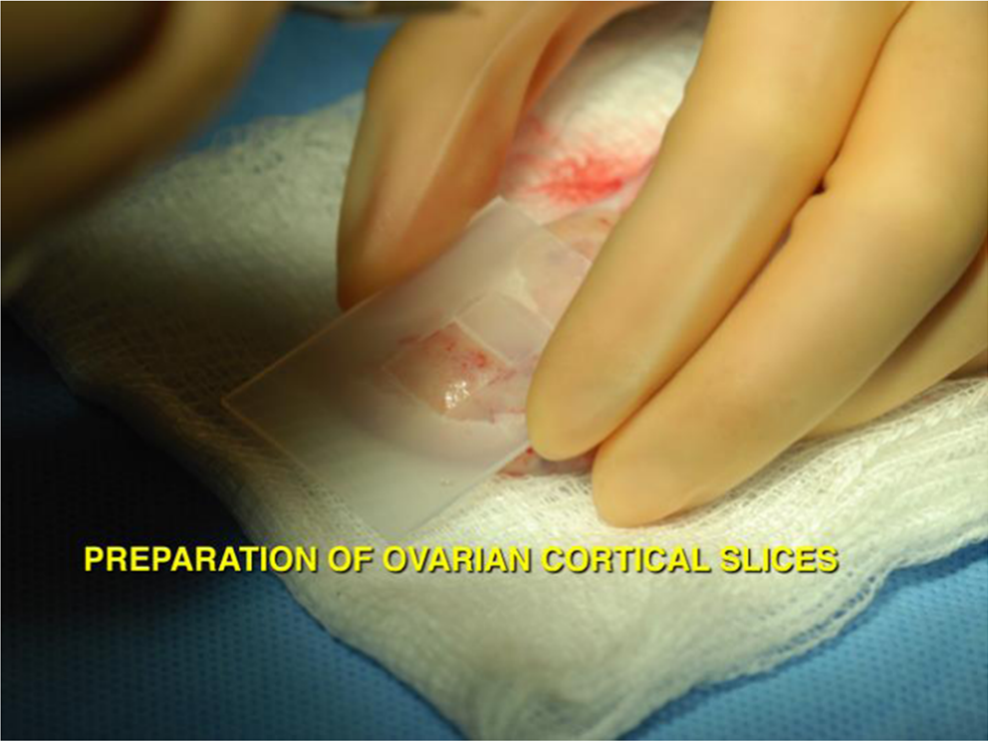
Thin slices of ovarian cortical tissue preserve all of the resting follicles

Using the vitrification technique, cortex tissue of each ovary is cut into slices 10 mm by 10 mm × 1 mm. Ovarian tissues are initially equilibrated in 7.5 % ethylene glycol (EG) and 7.5 % dimethyl sulfoxide (DMSO) in handling medium (HM: HEPES-buffered TCM-199 solution supplemented with 20 % serum for 25 min, followed by a second equilibration in 20 % EG and 20 % DMSO with 0.5 mol/l sucrose for 15 min). Ovarian tissues are then placed in a minimum volume of solution (virtually “dry”) onto a thin metal strip (Cryotissue: Kitazato BioPharma, Japan) and submerged directly into sterile liquid nitrogen [65], after which the strip is inserted into a protective container and placed into a liquid nitrogen storage tank (Fig. 8).

For thaw, the protective cover is removed and the Cryotissue metal strip is immersed directly into 40 ml of 37 °C HM solution supplemented with 1.0 mol/1 sucrose for 1 min. Then, ovary tissues are transferred into 40 ml of 0.5 mol/1 sucrose HM solution for 5 min at room temperature and washed twice in HM solution for 10 min before viability analysis or transplantation. No ice crystal formation occurs during any of these vitrification procedures [65, 66].

## Clinical benefit of ovarian tissue freezing: cancer, ovarian reserve, and long-term function

The most common benefit of ovarian transplant is not the unusual case of fresh grafting in identical twins but rather to protect the fertility and future endocrine function of young women undergoing treatment for cancer or other diseases that result in ovotoxicity. Since 1996, we have frozen ovary tissue for over 100 young women with cancer, or at risk for POF, of whom 16 had spare frozen tissue subjected to detailed viability testing before cryopreservation and after thaw.

**Fig. 9:**
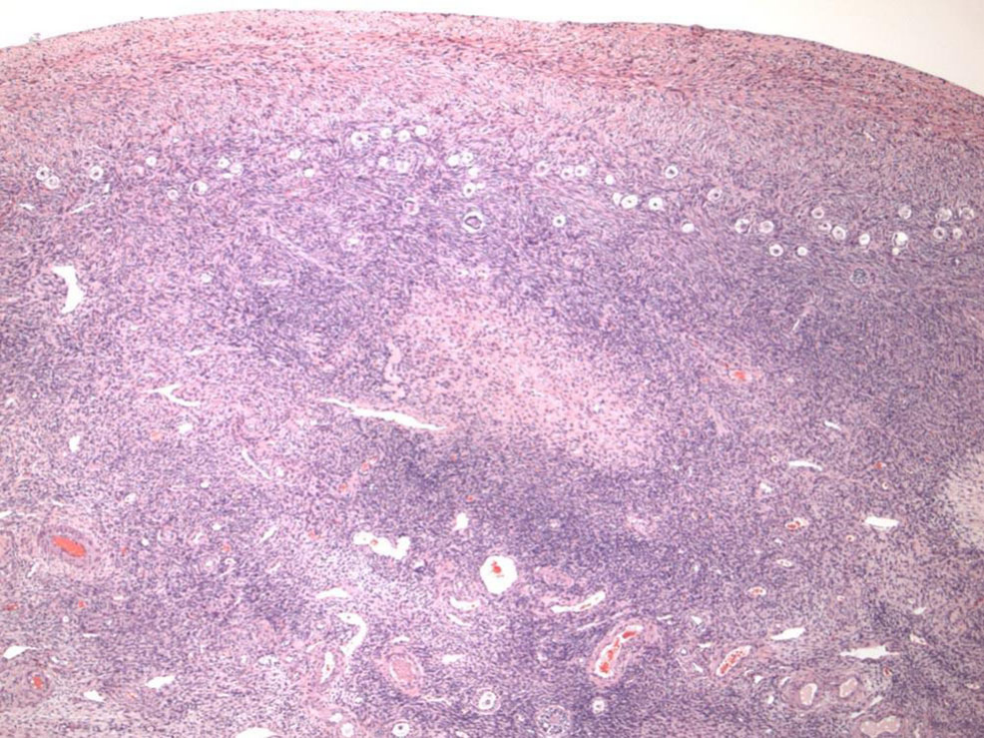
No metastasis in ovary

None of our cases who were cured of cancer have had any tumor cells in their ovary. There have been no cases reported of transmission of cancer via transplant of frozen ovarian cortex [77, 78, 80] (Fig. 9). The reason for the remarkable absence of ovarian metastasis might possibly be due to the fibrous avascular nature of the ovarian cortex [81]. The reason why fetal ovarian tubules (which in the male become seminiferous tubules) invade the fibrous cortex and become follicles is that the dense fibrous tissue of the cortex (which in the fetal and adult testis is just tunica albuginea) is needed to suppress the resting follicles from developing all at once prematurely by forming primordial follicles. Primordial follicles arrest the fetal oocytes from continuing meiosis to completion and subsequent apoptosis. The dense fibrous tissue of the ovarian cortex not only controls follicle development but also represents a relatively inhospitable location for cancer cells.

The return of FSH to normal at 4 to 5 months indicates that this is the period of time required for primordial follicles, once recruited, to develop on to the antral and ovulatory stage. The concomitant rise of AMH to well over normal levels followed by a drop to very low levels indicates a massive over-recruitment of follicles and subsequent depletion. This is substantiated by the current report of Winkler-Crepaz et al. transplanting human ovarian tissue into SCID mice, demonstrating no ischemic apoptosis of follicles, but rather a massive over-recruitment [36]. However, these transplanted slices of ovarian cortex continue to function normally for many years because of a decreased rate of primordial follicle recruitment that occurs when there is decreased ovarian reserve [82].

After ovarian transplantation, all patients were able to attempt natural conception every month without medical assistance. In fact, the commonly held view that egg freezing is a proven technique and ovary tissue transplantation is “experimental” is belied by the fact that most of the successful pregnancies resulting from fertility preservation in cancer patients thus far have been from frozen ovary tissue, and few at the date of this writing have come from frozen oocytes [77, 78].

At the time of this submission, we are aware of numerous other births after implanting ovarian tissue for a total of over 70 live births thus far [68]. To date, no reports of transmission of cancer from ovarian transplants have appeared (Fig. 9). One reason for the lack of transmission may be because the dense ovarian cortical fibrous tissue, like the tunica albuginea of the testis, is an inappropriate location for cancer cells to lodge.

Another plausible clinical benefit of the massive over-recruitment occurring after ovarian cortex transplantation is the recruitment of “trapped” follicles in menopausal ovaries [83, 84]. As we have shown, a reduced ovarian reserve results in a reduced rate of primordial follicle recruitment. The point at which the ovarian reserve is so low as to preclude recruitment, a state of POF is obtained (or ovarian POI), i.e., premature ovarian failure. We speculate that autotransplanting this tissue can result in recruitment and ovulation of these few remaining follicles.

## Primordial follicle recruitment and in vitro oogenesis

The ovarian cortex participates in the locking and the unlocking of the primordial follicle, and until the transplant is healed, we suspect this underlying mechanism to be disrupted. Intrinsic tissue pressure may be one mechanism at work to control primordial follicle status. The study by Winkler-Crepaz et al. provides evidence to suggest that the over-recruitment of follicles after ovarian cortical transplantation may be mediated by a biochemical mechanism via PTEN [36]. FOXO 3 translocation into the nucleus is already recognized as one mediator of primordial follicle arrest possibly induced by intrinsic pressure gradients established in the fibrous ovarian cortex.

Going beyond this, why should the oocyte begin meiosis and then be locked for a lifetime, while sperm are constantly produced by spermatogenic stem cells? What is the benefit to the species of such a dichotomy? The benefit of this dichotomy between spermatogenesis and oogenesis is that most of the mutations that occur in a species over many years occur during spermatogenesis in the testis, as “xeroxing” errors. The oocyte is spared that risk by not having to undergo recurrent mitosis. But of course the oocyte unfortunately ages, causing infertility. These two mechanisms are wedded to each other evolutionarily. Without the mutations caused by spermatogenesis and thus constant germ cell error-prone duplication, there would be no evolution. However, without the locking of the oocyte and avoidance of constant duplication errors as what occurs in the sperm, the species would have no stability.

The oocyte’s problem clinically is that it ages. The sperm’s problem is that it is constantly making errors during cell cycle duplication. Thus, in this view, the oocyte genetically stabilizes the species, while the sperm allows for evolution by encouraging the acquisition of a mutational load. These two opposing mechanisms have to strike a balance with each other. Men remain fertile as they age but threaten the genetic stability of the species, whereas women become infertile as they age but genetically stabilize the species. Primordial follicles were designed to resist oocyte development, in order to save the oocyte, and stabilize the species.

Primordial follicle arrest is the key to saving the oocyte from disappearing after the fetal initiation of meiosis and the continuation all the way through meiosis with subsequent apoptosis. It is also the key to the cautious gradual release every month in the young adult of an average of 1000 oocytes (30 per day) to develop over 4 months into gonadotropin-sensitive antral and Graffian follicles, sparing the resting oocytes from sudden total depletion, such as what occurs briefly after ovarian transplantation.

After this depletion of resting follicles is halted, the ovarian transplant then proceeds to function for years quite well despite a very low AMH and a low remaining number of follicles. That is because as the ovarian reserve goes down, the rate of primordial follicle recruitment in a compensatory way also goes down. That is why unilateral oophorectomy does not cause much of an earlier menopause (perhaps 1 year earlier in the human) [63]. The less the number of remaining oocytes, the better able primordial follicles are at maintaining their locking mechanism and, as a result, limiting the number of resting follicles allowed to activate and hence maintaining the follicle reserve [38–42].

## References

[1] Connolly MP, Pollard MS, Hoorens S, Kaplan BR, Oskowitz SP, Silber SJ (2008). Long-term economic benefits attributed to IVF-conceived children: a lifetime tax calculation. Am J Manag Care.

[2] Silber SJ. Are we infertile? Simpler treatments. In: How to get pregnant. Boston: Little, Brown; 2007. p. 87.

[3] Mosher WD, Pratt WF. Fecundity and infertility in the United States, 1965–82. Advance Data. 1985.

[4] Baerwald AR, Adams GP, Pierson RA (2012). Ovarian antral folliculogenesis during the human menstrual cycle: a review. Hum Reprod Update.

[5] Leridon H (2004). Can assisted reproduction technology compensate for the natural decline in fertility with age? A model assessment. Hum Reprod.

[6] Lampic C, Svanberg AS, Karlstrom P, Tyden T (2006). Fertility awareness, intentions concerning childbearing, and attitudes toward parenthood among female and male academics. Hum Reprod.

[7] Maheshwari A, Porter M, Shetty A, Bhattacharya S (2008). Women’s awareness and perceptions of delay in childbearing. Fertil Steril.

[8] te Velde ER, Pearson PL (2002). The variability of female reproductive ageing. Hum Reprod Update.

[9] Devroey P (1996). Female age predicts embryonic implantation after ICSI: a case-controlled study. Hum Reprod.

[10] Silber SJ, Nagy Z, Devroey P, Camus M, Van Steirteghem AC (1997). The effect of female age and ovarian reserve on pregnancy rate in male infertility: treatment of azoospermia with sperm retrieval and intracytoplasmic sperm injection. Hum Reprod.

[11] Fretts RC. Effect of advanced age on fertility and pregnancy in women. Up To Date Online 2007. Available at: http://www.uptodate.com.

[12] SART. Assisted reproductive technology success rates. National summary and fertility clinic reports. Atlanta, GA: Centers for Disease Control and Prevention, 2005.

[13] Bleyer WA (1990). The impact of childhood cancer on the United States and the world. CA Cancer J Clin.

[14] Ries LAG. Cancer incidence and survival among children and adolescents: United States SEER program 1975–1995. 1999.

[15] Jeruss JS, Woodruff TK (2009). Preservation of fertility in patients with cancer. N Engl J Med.

[16] Anderson RA, Themmen AP, Al-Qahtani A, Groome NP, Cameron DA (2006). The effects of chemotherapy and long-term gonadotrophin suppression on the ovarian reserve in premenopausal women with breast cancer. Hum Reprod.

[17] Anderson RA, Cameron DA (2007). Assessment of the effect of chemotherapy on ovarian function in women with breast cancer. J Clin Oncol.

[18] Larsen EC, Muller J, Schmiegelow K, Rechnitzer C, Andersen AN (2003). Reduced ovarian function in long-term survivors of radiation and chemotherapy-treated childhood cancer. J Clin Endocrinol Metab.

[19] Lee SJ, Schover LR, Partridge AH, Patrizio P, Wallace WH, Hagerty K (2006). American Society of Clinical Oncology recommendations on fertility preservation in cancer patients. J Clin Oncol.

[20] Schover LR, Rybicki LA, Martin BA, Bringelsen KA (1999). Having children after cancer. A pilot survey of survivors’ attitudes and experiences. Cancer.

[21] Schover LR, Brey K, Lichtin A, Lipshultz LI, Jeha S (2002). Knowledge and experience regarding cancer, infertility, and sperm banking in younger male survivors. J Clin Oncol.

[22] Gosden RG, Baird DT, Wade JC, Webb R (1994). Restoration of fertility to oophorectomized sheep by ovarian autografts stored at −196 degrees C. Hum Reprod.

[23] Gook DA, Edgar DH, Stern C (1999). Effect of cooling rate and dehydration regimen on the histological appearance of human ovarian cortex following cryopreservation in 1, 2-propanediol. Hum Reprod.

[24] Newton H, Aubard Y, Rutherford A, Sharma V, Gosden R (1996). Low temperature storage and grafting of human ovarian tissue. Hum Reprod.

[25] Homburg R, van der Veen F, Silber SJ (2009). Oocyte vitrification—women’s emancipation set in stone. Fertil Steril.

[26] Kuwayama M, Vajta G, Kato O, Leibo SP (2005). Highly efficient vitrification method for cryopreservation of human oocytes. Reprod Biomed Online.

[27] Cobo A, Garcia-Velasco JA, Domingo J, Remoh J, Pellicer A (2013). Is vitrification of oocytes useful for fertility preservation for age-related fertility decline and in cancer patients?. Fertil Steril.

[28] Cobo A, Diaz C (2011). Clinical application of oocyte vitrification: a systematic review and meta-analysis of randomized controlled trials. Fertil Steril.

[29] Cobo A, Vajta G, Remoh J (2009). Vitrification of human mature oocytes in clinical practice. Reprod Biomed Online.

[30] Smith GD, Serafini PC, Fioravanti J (2010). Prospective randomized comparison of human oocyte cryopreservation with slow-rate freezing or vitrification. Fertil Steril.

[31] Cobo A, Kuwayama M, Perez S, Ruiz A, Pellicer A, Remoh J (2008). Comparison of concomitant outcome achieved with fresh and cryopreserved donor oocytes vitrified by the Cryotop method. Fertil Steril.

[32] Rienzi L, Cobo A, Paffoni A (2012). Consistent and predictable delivery rates after oocyte vitrification: an observational longitudinal cohort multicentric study. Hum Reprod.

[33] Cil AP, Bang H, Oktay K (2013). Age-specific probability of live birth with oocyte cryopreservation: an individual patient data meta-analysis. Fertil Steril.

[34] Stoop D, Maes E, Polyzos NP, Verheyen G, Tournaye H, Nekkebroeck J (2014). Oocyte banking for anticipated gamete exhaustion (AGE) is a preventive intervention, neither social nor nonmedical. Reprod Biomed Online.

[35] Patrizio P, Sakkas D (2009). From oocyte to baby: a clinical evaluation of the biological efficiency of in vitro fertilization. Fertil Steril.

[36] Winkler-Crepaz et al. Follicular growth after xenotransplantation of cryopreserved/thawed human ovarian tissue in SCID mice: dynamic and molecular aspects. J Assist Reprod Genet. 2016. 10.1007/s10815-016-0769-2. 10.1007/s10815-016-0769-2 PMC5171895 27465301

[37] Silber SJ, Lenahan KM, Levine DJ (2005). Ovarian transplantation between monozygotic twins discordant for premature ovarian failure. N Engl J Med.

[38] Silber S (2015). Unifying theory of adult resting follicle recruitment and fetal oocyte arrest. Reprod Biomed Online.

[39] Silber S, Pineda J, Lenahan K, DeRosa M, Melnick J (2015). Fresh and cryopreserved ovary transplantation and resting follicle recruitment. Reprod Biomed Online.

[40] Hayashi K, Ogushi S, Kurimoto K, Shimamoto S, Ohta H, Saitou M (2012). Offspring from oocytes derived from in vitro primordial germ cell-like cells in mice. Science.

[41] Hayashi K, Ohta H, Kurimoto K, Aramaki S, Saitou M (2011). Reconstitution of the mouse germ cell specification pathway in culture by pluripotent stem cells. Cell.

[42] Hayashi K, Saitou M (2013). Generation of eggs from mouse embryonic stem cells and induced pluripotent stem cells. Nat Protoc.

[43] Gunasena KT, Villines PM, Critser ES, Critser JK (1997). Live births after autologous transplant of cryopreserved mouse ovaries. Hum Reprod.

[44] Deanesly R (1954). Immature rat ovaries grafted after freezing and thawing. J Endocrinol.

[45] Parrott DMV (1960). The fertility of mice with orthotopic ovarian grafts derived from frozen tissue. J Reprod Fertil.

[46] Candy CJ, Wood MJ, Whittingham DG (2000). Restoration of a normal reproductive lifespan after grafting of cryopreserved mouse ovaries. Hum Reprod.

[47] Aubard Y, Piver P, Cogni Y, Fermeaux V, Poulin N, Driancourt MA (1999). Orthotopic and heterotopic autografts of frozen-thawed ovarian cortex in sheep. Hum Reprod.

[48] Donnez J, Dolmans MM, Demylle D (2004). Live birth after orthotopic transplantation of cryopreserved ovarian tissue. Lancet.

[49] Meirow D, Levron J, Eldar-Geva T (2005). Pregnancy after transplantation of cryopreserved ovarian tissue in a patient with ovarian failure after chemotherapy. N Engl J Med.

[50] Silber SJ, Gosden RG (2007). Ovarian transplantation in a series of monozygotic twins discordant for ovarian failure. N Engl J Med.

[51] Silber SJ, DeRosa M, Pineda J, Lenahan K, Grenia D, Gorman K (2008). A series of monozygotic twins discordant for ovarian failure: ovary transplantation (cortical versus microvascular) and cryopreservation. Hum Reprod.

[52] Silber SJ, Grudzinskas G, Gosden RG (2008). Successful pregnancy after microsurgical transplantation of an intact ovary. N Engl J Med.

[53] Gosden RG, Telfer E, Faddy MJ, Brook DJ (1989). Ovarian cyclicity and follicular recruitment in unilaterally ovariectomized mice. J Reprod Fertil.

[54] Faddy MJ, Gosden RG, Gougeon A, Richardson SJ, Nelson JF (1992). Accelerated disappearance of ovarian follicles in mid-life: implications for forecasting menopause. Hum Reprod.

[55] Aydin Y, Celiloglu M, Koyuncuoglu M, Ulukus C (2010). Follicular dynamics and apoptosis following unilateral oophorectomy. Syst Bio Reprod Med.

[56] Kaaijk EM, Hamerlynck JV, Beek JF, van der Veen F (1999). Clinical outcome after unilateral oophorectomy in patients with polycystic ovary syndrome. Hum Reprod.

[57] Kim SS (2003). Ovarian tissue banking for cancer patients. To do or not to do?. Hum Reprod.

[58] Koskas M, Uzan C, Gouy S (2011). Fertility determinants after conservative surgery for mucinous borderline tumours of the ovary (excluding peritoneal pseudomyxoma). Hum Reprod.

[59] Meredith S, Dudenhoeffer G, Butcher RL, Lerner SP, Walls T (1992). Unilateral ovariectomy increases loss of primordial follicles and is associated with increased metestrous concentration of follicle stimulating hormone in old rats. Biol Reprod.

[60] Saiduddin S, Rowe RF, Casida LE (1970). Ovarian follicular changes following unilateral ovariectomy in the cow. Biol Reprod.

[61] Thomas-Teinturier C, El Fayech C, Oberlin O (2013). Age at menopause and its influencing factors in a cohort of survivors of childhood cancer: earlier but rarely premature. Hum Reprod.

[62] Zhai A, Axt J, Hamilton EC, Koehler E, Lovvorn HN (2012). Assessing gonadal function after childhood ovarian surgery. J Pediatr Surg.

[63] Yasui T, Hayashi K, Mizunuma H (2012). Factors associated with premature ovarian failure, early menopause and earlier onset of menopause in Japanese women. Maturitas.

[64] Keros V, Xella S, Hultenby K (2009). Vitrification versus controlled-rate freezing in cryopreservation of human ovarian tissue. Hum Reprod.

[65] Kagawa N, Silber S, Kuwayama M (2009). Successful vitrification of bovine and human ovarian tissue. Reprod Biomed Online.

[66] Silber S, Kagawa N, Kuwayama M, Gosden R (2010). Duration of fertility after fresh and frozen ovary transplantation. Fertil Steril.

[67] Stoop D, Cobo A, Silber S (2014). Fertility preservation for age-related fertility decline. Lancet.

[68] Donnez J, Dolmans MM (2015). Ovarian cortex transplantation: 60 reported live births brings the success and worldwide expansion of the technique towards routine clinical practice. J Assist Reprod Genet.

[69] Andersen CY, Rosendahl M, Byskov AG (2008). Two successful pregnancies following autotransplantation of frozen/thawed ovarian tissue. Hum Reprod.

[70] Revel A, Laufer N, Ben Meir A, Lebovich M, Mitrani E (2011). Micro-organ ovarian transplantation enables pregnancy: a case report. Hum Reprod.

[71] Revelli A, Marchino G, Dolfin E (2013). Live birth after orthotopic grafting of autologous cryopreserved ovarian tissue and spontaneous conception in Italy. Fertil Steril.

[72] Dittrich R, Lotz L, Keck G (2012). Live birth after ovarian tissue autotransplantation following overnight transportation before cryopreservation. Fertil Steril.

[73] Andersen CY, Silber SJ, Bergholdt SH, Jorgensen JS, Ernst E (2012). Long-term duration of function of ovarian tissue transplants: case reports. Reprod Biomed Online.

[74] Demeestere I, Simon P, Emiliani S, Delbaere A, Englert Y (2007). Fertility preservation: successful transplantation of cryopreserved ovarian tissue in a young patient previously treated for Hodgkin’s disease. Oncologist.

[75] Sanchez-Serrano M, Crespo J, Mirabet V (2010). Twins born after transplantation of ovarian cortical tissue and oocyte vitrification. Fertil Steril.

[76] Piver P, Amiot C, Agnani G (2009). Two pregnancies obtained after a new technique of autotransplantation of cryopreserved ovarian tissue. Hum Reprod.

[77] Donnez J, Dolmans MM, Pellicer A (2013). Restoration of ovarian activity and pregnancy after transplantation of cryopreserved ovarian tissue: a review of 60 cases of reimplantation. Fertil Steril.

[78] Jensen AK, Kristensen SG, Macklon KT (2015). Outcomes of transplantations of cryopreserved ovarian tissue to 41 women in Denmark. Hum Reprod.

[79] Gook DA, Edgar DH, Stern C (2000). The effects of cryopreservation regimens on the morphology of human ovarian tissue. Mol Cell Endocrinol.

[80] Greve T, Clasen-Linde E, Andersen MT (2012). Cryopreserved ovarian cortex from patients with leukemia n complete remission contains no apparent viable malignant cells. Blood.

[81] Ortega JJ, Javier G, Toran N (1981). Testicular relapses in childhood acute lymphoid leukaemia (author’s transl). Sangre.

[82] Wallace WH, Kelsey TW (2010). Human ovarian reserve from conception to the menopause. PLoS One.

[83] Kawamura K, Cheng Y, Suzuki N (2013). Hippo signaling disruption and Akt stimulation of ovarian follicles for infertility treatment. Proc Natl Acad Sci U S A.

[84] Suzuki N, Yoshioka N, Takae S (2015). Successful fertility preservation following ovarian tissue vitrification in patients with primary ovarian insufficiency. Hum Reprod.

